# Interpreting hand grip strength in hospital employees with post-COVID syndrome compared to non-infected controls: a case-control study

**DOI:** 10.1038/s41598-026-51666-w

**Published:** 2026-05-09

**Authors:** Matthias Tack, Rosalie Gruber, Leia Betting, Swetlana Herbrandt, Gerlinde Schlang, Frauke Mattner

**Affiliations:** 1 Institute of Hygiene, Cologne Merheim Medical Center, University of Witten/Herdecke, Ostmerheimerstr. 200, 51109 Köln, Germany; 2 Statistical Consulting and Analysis, Center for Higher Education, TU Dortmund University, Dortmund, Germany; 3 Department of Occupational Medicine, Cologne Merheim Medical Center, Cologne, Germany

**Keywords:** SARS-CoV-2, Myalgic encephalomyelitis/chronic fatigue syndrome (ME/CFS), Post-COVID syndrome, Healthcare worker, Hand grip strength, Post-exertional malaise, Diseases, Health care, Medical research

## Abstract

Post-COVID syndrome (PCS) is characterized by a variety of persistent symptoms following SARS-CoV-2 infection, including fatigue among others. Myalgic encephalomyelitis/chronic fatigue syndrome (ME/CFS) is a related neurological disorder primarily characterized by severe fatigue and post-exertional malaise. This exploratory study aimed to assess hand grip strength (HGS) in individuals with PCS to evaluate muscular performance and fatigability and to explore potential HGS-derived parameters associated with PCS. HGS was measured in 19 hospital employees with PCS (mean age 47.8; 89.5% female; 7 fulfilling ME/CFS criteria) and compared with 23 healthy controls (mean age 43.7; 69.6% female). Measurements were performed in two sessions separated by 60 min, each consisting of ten consecutive HGS measurements. Linear mixed model analysis indicated that HGS tended to be lower in PCS at specific measurement points, although no consistent overall group effect was observed. HGS was reduced in the second session in PCS but not in controls, suggesting possible alterations in recovery following repeated exertion. Exploratory analysis of 30 HGS-derived parameters using logisitic regression models in female participants identified parameters based on maximum, minimum, and mean force values as showing the most promising discriminatory patterns: however, predictive performace was moderate and should be interpreted with caution. Overall, HGS may provide insights into funcitional impairment in PCS and could serve as a supportive adjunct in clinical assessment, although its diagnostic utility requires validation in larger cohorts.

**Supplementary Information:**

The online version contains supplementary material available at 10.1038/s41598-026-51666-w.

## Introduction

Following an infection with severe acute respiratory syndrome coronavirus (SARS-CoV-2), 5–10% of individuals develop long-lasting symptomatology with high individual health burdens^1,2^. The term long COVID refers to persistent or newly emerging symptoms following acute SARS-CoV-2 infection beyond four weeks^3^. Within this spectrum, the World Health Organization (WHO) defines post COVID-19 condition or post-COVID syndrome (PCS) as symptoms occurring three months after probable or confirmed SARS-CoV-2 infection, lasting for at least two months, and not explained by alternative diagnoses^4^. Symptom clusters include persistent fatigue, neurocognitive impairment, chest pain and shortness of breath^5^. PCS is a multisystemic condition with several pathomechanisms that are not yet well understood^6^.

A subset of PCS patients fullfill diagnostic criteria for myalgic encephalomyelitis/chronic fatigue syndrome (ME/CFS)^7,8^. ME/CFS is a complex and debilitating neurological disease limiting daily activities and quality of life^8–10^. In most cases, patients report preceding infections or infection-like symptomatology; however, other triggers such as surgical procedures have also been described, and in some cases no clear initiating event can be identified^8,11,12^. The worldwide prevalence was estimated to be 0.3–0.9% before the pandemic, with a significant increase expected during the COVID-19 pandemic, and at least two thirds of cases occuring in females^8,13^. The most prominent symptom is post-exertional malaise (PEM), an exceeding muscular and cognitive fatigability after cognitive, emotional or light physical activity, lasting days to weeks^9,14^. Due to the absence of specific biomarkers, diagnosis relies on clinical evaluation using the Canadian Consensus Criteria (CCC), and no curative treatment has been established to date; management remains symptomatic^8,15^. Dysregulated involvement of autonomic and central nervous, immune and cardiovascular system, viral replication, dysfunction of cellular energy metabolism and endothelium are probable causes^9,15,16^.

Both conditions rely on subjective reports and clinical criteria for diagnosis as well as classification of impairment. Assessment of hand grip strength (HGS) with dynamometers has been used as a reliable tool, to quantify muscular strength and fatigue as an objective marker with high test-retest reliability (intraclass correlation coefficient 0.92–0.96)^17^. It has been used in musculoskelatal, neurological conditions or general assessment of physical performance^17,18^. Different studies demonstrated impaired HGS in ME/CFS patients^19,20^. Additionally, lower HGS was correlated with higher levels of PEM, fatigue and pain^19,20^. HGS determination enables more insides in the underlying pathophysiology as studies show impaired recovery in ME/CFS patients^19–21^. More recently HGS has been applied to PCS patients^7,22^. A European expert consensus recommends HGS determination as an assessment tool in ME/CFS patients^16^, however clinical protocols for its use are not established.

In this study, we aimed to compare HGS in hospital employees (HE) with PCS and fatigue as a leading symptom to healthy controls (HC) with no documented SARS-CoV-2 infection in order to identify characteristic differences in force performance and fatigability. To this end, HGS trajectories across repeated measurements and sessions were analyzed using a linear mixed model to assess group differences while accounting for relevant covariates and within-subject variability. In addition, an exploratory set of established and newly derived HGS parameters was evaluated to identify those most suitable for distinguishing between PCS and control participants. The predictive value of these parameters for group classification was assessed using logistic and mixed-effects logistic regression models with bootstrap-based validation.

## Results

In total, 42 individuals were recruited for the HGS measurement, including 19 HEs with PCS (hereafter referred to as the PCS group) and 23 HCs with no known history of SARS-CoV-2 infection based on testing history and symptom assessment. Females were overrepresented in both groups (PCS 89.5% and HC 69.6%). The PCS group was slightly older on average (47.8 years) compared to the HC group (43.7 years). In both groups, the proportion of nursing staff was highest, followed by other professions (physiotherapists, support staff) and medical staff. The CCC for the diagnosis of ME/CFS were fulfilled in 7 participants in the PCS group and in none of the HC group. Functional status, assessed by the Bell Score, was lower in the PCS group compared to HC (mean 65.3 vs. 93.9 points). Further characteristics are presented in Table 1. The assessment of the PCS group took place between July and December 2022, at a median of 629 days (467 − 1008 days) after the first SARS-CoV-2 infection. According to WHO criteria, all PCS participants had experienced mild to moderate COVID-19, and none required hospitalization during the acute phase^23^. All participants in the PCS group were unvaccinated at the time of their initial infection, as vaccines were not yet available. At the time of assessment, participants had received COVID-19 vaccinations; the number of doses is summarized in Table 1. Based on epidemiological data on circulating SARS-CoV-2 variants in Germany and the federal state of North Rhine-Westphalia during the respective infection period, the PCS group was most likely infected with wild-type SARS-CoV-2 or the Alpha variant (B.1.1.7)^24^. Following the initial infection, 42% of participants of the PCS group reported at least one SARS-CoV-2 reinfection, with a total of one to five infections per individual until the time of assessment.

**Table 1:** Characteristics of the study population.

	PCS		HC	
Total number (n)	19 (100)		23 (100)	
Demographics				
Females (n)	17 (89.5)		16 (69.6)	
Males (n)	2 (10.5)		7 (30.4)	
Age (years)	47.8 ± 10.8 (25–61)		43.7 ± 12.0 (22–59)	
Professional group				
Nursing staff (n)	14 (73.7)		10 (43.5)	
Medical staff (n)	1 (5.3)		4 (17.4)	
Others (n)	4 (21.1)		9 (39.1)	
SARS-CoV-2 infection				
Yes (n)	19 (100)		0 (0.0)	
Presumable not (n)	0 (0.0)		23 (100)	
SARS-CoV-2 reinfection				
1–2 infections in total	17 (89.5)		-	
≥ 3 infections in total	2 (10.5)		-	
COVID-19 vaccinations at time of initial SARS-CoV-2 infection				
Unvaccinated at first SARS-CoV-2 infection (n)	19 (100)		-	
COVID-19 vaccinations at time of assessment				
1–2 vaccinations (n)	10 (52.6)		2 (8.7)	
3–4 vaccinations (n)	8 (42.1)		20 (87.0)	
NA (n)*	1 (5.3)		1 (4.3)	
Bell Score 1995				
Bell Score (points)	65.3 ± 12.6 (40–90)		93.9 ± 13.4 (50–100)	
CCC				
Fulfilled (n)	7 (36.8)		0 (0.0)	
Not fulfilled (n)	12 (63.2)		23 (100)	
Total points	16.4 ± 6.9 (8–33)		2.0 ± 3.1 (0–12)	
Comorbidity				
Arterial hypertension	7 (36.8)		1 (4.3)	
Asthma/COPD	5 (26.3)		0 (0.0)	
Thyroid disorders	4 (21.1)		0 (0.0)	
Type 2 diabetes mellitus	3 (15.8)		0 (0.0)	
Rheumatoid arthritis	0 (0.0)		1 (0.0)	
Obstructive sleep apnea	4 (21.1)		0 (0.0)	
No reported comorbidities	6 (31.6)		21 (91.3)	

*NA indicates missing data regarding the number of COVID-19 vaccinations; vaccination was reported, but the exact number was unavailable.

Categorial variables are expressed as numbers (n) and percentages in brackets. Continuous variables are expressed as mean values ± standard deviation and extreme values (range) in brackets. All reported comorbidities were under medical treatment and considered clinically stable at the time of assessment. CCC, Canadian Census Criteria; COPD, Chronic Obstructive Pulmonary Disease; HC, healthy controls; PCS, post-COVID syndrome.

### Hand grip strength measurements

**Fig. 1:**
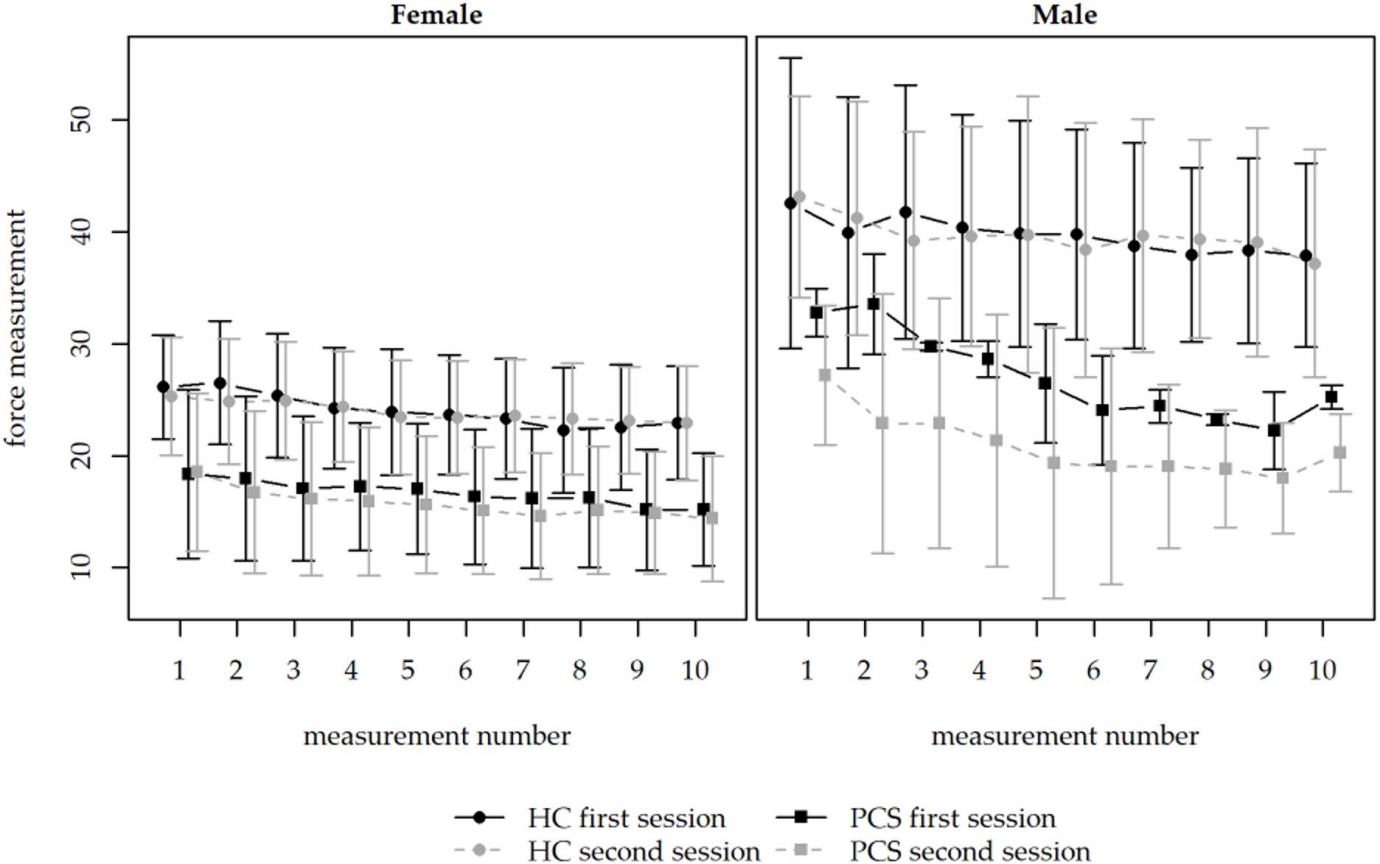
Comparison of hand grip strength (HGS) in hospital employees with post-COVID syndrome (PCS; squares; *n* = 19) to hospital employees as healthy controls (HC; circles; *n* = 23) in two sessions. Both sessions comprised ten consecutive measurements of maximal HGS with an electronic hand dynamometer with an interval of one hour in between. Graphic respresentation shows lower HGS of PCS in females and males. Left: female PCS (circles, *n* = 17), HC (squares, *n* = 16); right: male PCS (circles, *n* = 2) and HC (squares, *n* = 7). Continuous line: first session, dotted line: second session. Mean values in kg with standard deviation.

As visualized in Fig. 1, participants with PCS demonstrated lower HGS in both females and males compared with HC. HGS declined across all 10 measurements during the first and second sessions. A decline between the first and second session was observed in the PCS group.

### Linear mixed model analysis

A linear mixed model was used to analyze and compare HGS measurements between study groups. The estimated fixed effects are provided in Supplementary Table S1. A Global F-test of the model showed significant main effects of session (*p* < 0.001), measurement number (*p* < 0.001), age (*p* = 0.025), and sex (*p* < 0.001). No significant main effect of the study group was observed (*p* = 0.067). Interaction effects were not statistically significant, except for the interaction between age and sex (*p* = 0.024; Supplementary Table S2).

**Fig. 2:**
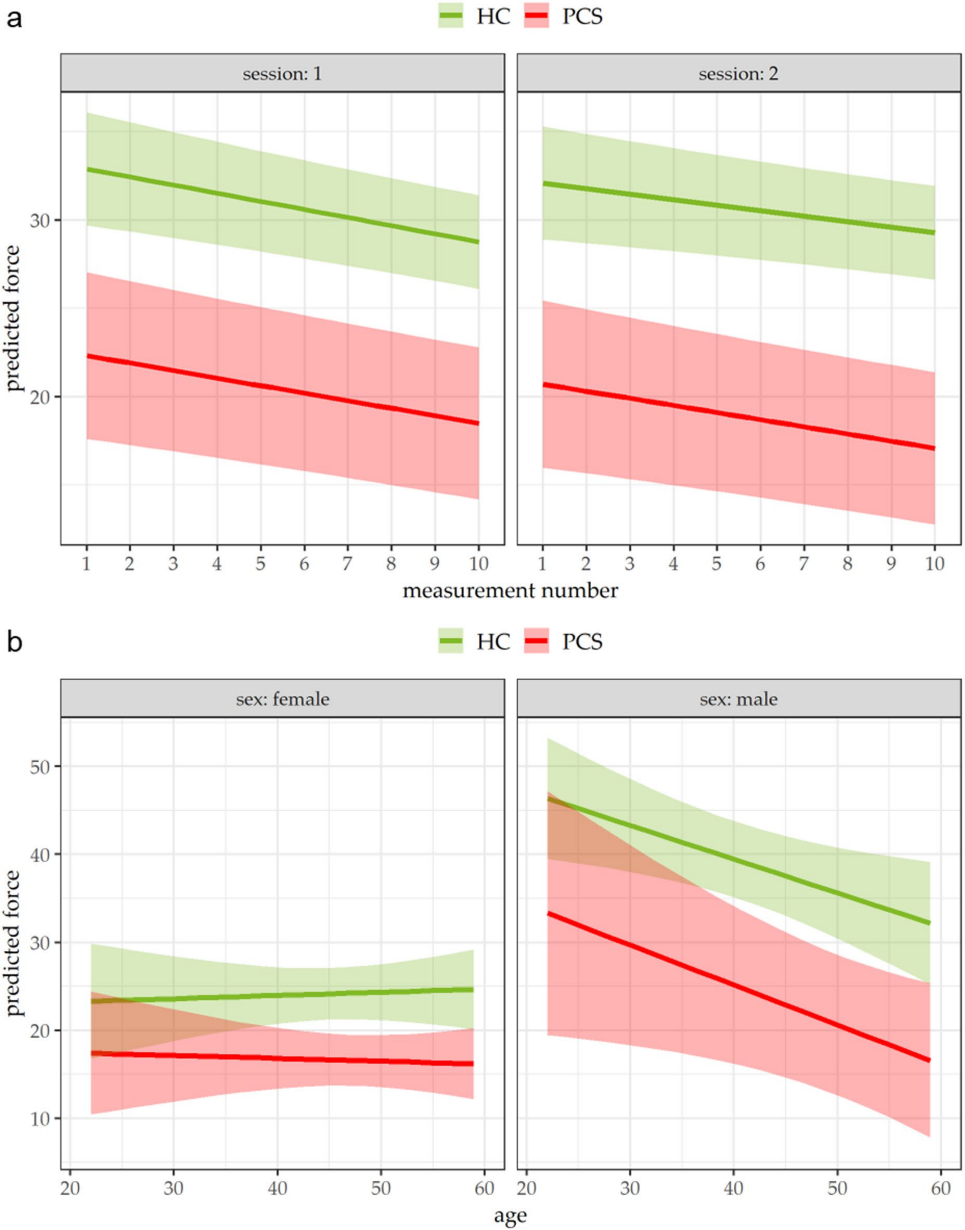
Linear mixed model prediction for HGS in hospital employees (HE) with post-COVID syndrome (PCS, red, *n* = 19) and HEs serving as healthy controls (HC, green, *n* = 23). Visualized is the model prediction for (**a**) the HGS in each session in the relation to the measurement number and (**b**) the HGS per sex in relation to age. Each sessions comprised ten consecutive measurements of maximal HGS with an electronic hand dynamometer with an interval of one hour in between. Mean values of HGS are visualized as colored lines with 95% confidence intervals for each measurement number and age point.

Predicted HGS differed significantly between PCS and control groups at specific measurement points. In session 1, differences were observed at measurement number 1 (estimate = − 10.57, *p* = 0.002) and 10 (estimate = − 10.27, *p* = 0.001), and in session 2 at measurement numbers 1 (estimate = − 11.39, *p* < 0.001) and 10 (estimate = − 12.21, *p* < 0.001). Across sessions, predicted HGS for the PCS group was lower at the beginning and end of each session (*p* < 0.003 and *p* < 0.013, respectively), whereas no significant session-related differences were observed in the control group (*p* > 0.05). Overall, HGS decreased from measurement 1 to 10 in both sessions and both study groups (*p* < 0.01, Table 2). The slopes of HGS decline did not differ significantly between sessions, study groups, or their interactions (*p* > 0.05, Supplementary Table S3). As illustrated in Fig. 2, predicted HGS was consistently lower in the PCS group compared with controls, with slightly reduced HGS in session 2 relative to session 1 for both groups.

**Table 2:** Comparison of hand grip strength prediction in a linear mixed model.

Comparison of force prediction for both study groups in relation to the session and measurement number									
Meas. no	Session	Study group	Estimate	SE	df	t ratio	*p* value	2.5%	97.5%
1	1	**PCS - HC**	−10.571	2.860	44.820	−3.696	**0.002**	−18.015	−3.127
	2	**PCS - HC**	−11.387	2.860	44.820	−3.981	**<0.001**	−18.830	−3.943
10	1	**PCS - HC**	−10.274	2.533	36.467	−4.057	**0.001**	−16.928	−3.619
	2	**PCS - HC**	−12.208	2.533	36.467	−4.820	**<0.001**	−18.863	−5.553
Comparison of force prediction for both sessions in relation to the study group and measurement number									
*meas. no*	*study group*	***session***	*estimate*	*SE*	*df*	*t ratio*	*p value*	*2.5%*	*97.5%*
1	HC	**2 − 1**	−0.800	0.437	752.000	−1.832	0.269	−1.893	0.293
	PCS	**2 − 1**	−1.616	0.480	752.000	−3.363	**0.003**	−2.818	−0.413
10	HC	**2 − 1**	0.516	0.437	752.000	1.183	0.949	−0.577	1.610
	PCS	**2 − 1**	−1.418	0.480	752.000	−2.952	**0.013**	−2.621	−0.215
Comparison of force prediction for the first and last measurement in relation to the study group and session									
*session*	*study group*	***meas. no***	*estimate*	*SE*	*df*	*t ratio*	*p value*	*2.5%*	*97.5%*
1	*HC*	**10 − 1**	−4.136	0.854	60.166	−4.844	**<0.001**	−6.335	−1.937
	PCS	**10 − 1**	−3.839	0.940	60.166	−4.086	**<0.001**	−6.258	−1.420
2	HC	**10 − 1**	−2.820	0.854	60.166	−3.302	**0.006**	−5.019	−0.621
	PCS	**10 − 1**	−3.641	0.940	60.166	−3.876	**0.001**	−6.061	−1.222
Comparison of force prediction for the both study groups in relation to sex and two age points									
*age*	*sex*	***study group***	*estimate*	*SE*	*df*	*t ratio*	*p value*	*2.5%*	*97.5%*
25	female	**PCS - HC**	−6.104	4.088	37.188	−1.493	0.575	−16.834	4.626
	male	**PCS - HC**	−13.264	6.093	36.101	−2.177	0.144	−29.282	2.754
55	female	**PCS - HC**	−8.186	2.501	38.386	−3.273	**0.009**	−14.740	−1.631
	male	**PCS - HC**	−15.346	4.816	36.673	−3.186	**0.012**	−27.998	−2.694
Comparison of force prediction for sex in relation to two age points and both study groups									
*study group*	*age*	***sex***	*estimate*	*SE*	*df*	*t ratio*	*p value*	*2.5%*	*97.5%*
*HC*	25	**male - female**	21.796	4.012	35.000	5.432	**<0.001**	11.230	32.362
	55	**male - female**	9.177	3.415	35.000	2.687	**0.044**	0.185	18.169
PCS	25	**male - female**	14.636	6.057	35.000	2.416	0.084	−1.316	30.587
	55	**male - female**	2.016	4.252	35.000	0.474	1.000	−9.182	13.215
Comparison of force prediction for two age points in relation to sex and both study groups									
*study group*	*sex*	***age***	*estimate*	*SE*	*df*	*t ratio*	*p value*	*2.5%*	*97.5%*
*HC*	female	**55 − 25**	1.103	3.730	35.000	0.296	1.000	−8.719	10.926
	male	**55 − 25**	−11.516	4.339	35.000	−2.654	**0.048**	−22.943	−0.088
PCS	female	**55 − 25**	−0.978	3.684	35.000	−0.266	1.000	−10.681	8.724
	male	**55 − 25**	−13.598	5.971	35.000	−2.277	0.116	−29.321	2.126

Post-hoc-tests comparing predicted hand grip strength (HGS) between hospital employees (HE) with post-COVID syndrome (PCS, *n* = 19) and HEs serving as healthy controls (HC, *n* = 23) using a linear mixed model. Variables compared are highlighted in bold and correspond to the first two columns of the table, indicating the factors or measurement points from the model. The 95% confidence intervals for the estimates are provided at the 2.5% and 97.5% limits. All p-values were Bonferroni-adjusted. SE, standard error; df, degrees of freedom.

For the analysis of age as a factor, predicted HGS was compared at 25 and 55 years. Between study groups, significant differences were observed at age 55 for both females (estimate = − 8.19, *p* = 0.009) and males (estimate = − 15.35, *p* = 0.012), whereas no significant differences were detected at age 25 (*p* > 0.05). Within the control group, predicted HGS differed significantly between females and males at both ages (25 years: estimate = 21.80, *p* < 0.001; 55 years: estimate = 9.18, *p* = 0.044), whereas no significant sex differences were observed within the PCS group. In addition, within the control group, predicted HGS differed significantly for males between ages 25 and 55 (estimate = − 11.52, *p* = 0.048, Table 2). Finally, the slopes of predicted HGS over the measurement sequence differed significantly between sexes, with a steeper decline observed in males compared to females (*p* = 0.024, Supplementary Table S3).

These results demonstrate that significant contrasts arise from group-by-measurement, group-by-session, and age- or sex-specific effects, while the overall main effect of the study group in the linear mixed model remains non-significant.

The model explained 94.3% of the total variance (conditional R^2^ = 0.943), with fixed effects accounting for 62.1% and random effects for 32.2% (Supplementary Figure S1). The root mean squared error (RMSE) for marginal predictions (6.084) was higher than for conditional predictions (2.401).

Sensitivity analyses using different specifications of the linear mixed model demonstrated that the estimated effect of the study group was reduced after adjustment for age and sex. The direction of the effect remained consistent across model specifications, and statistical significance was no longer observed in the fully adjusted model (Supplementary Table S4).

### Predictive modeling of PCS based on HGS parameters

**Fig. 3:**
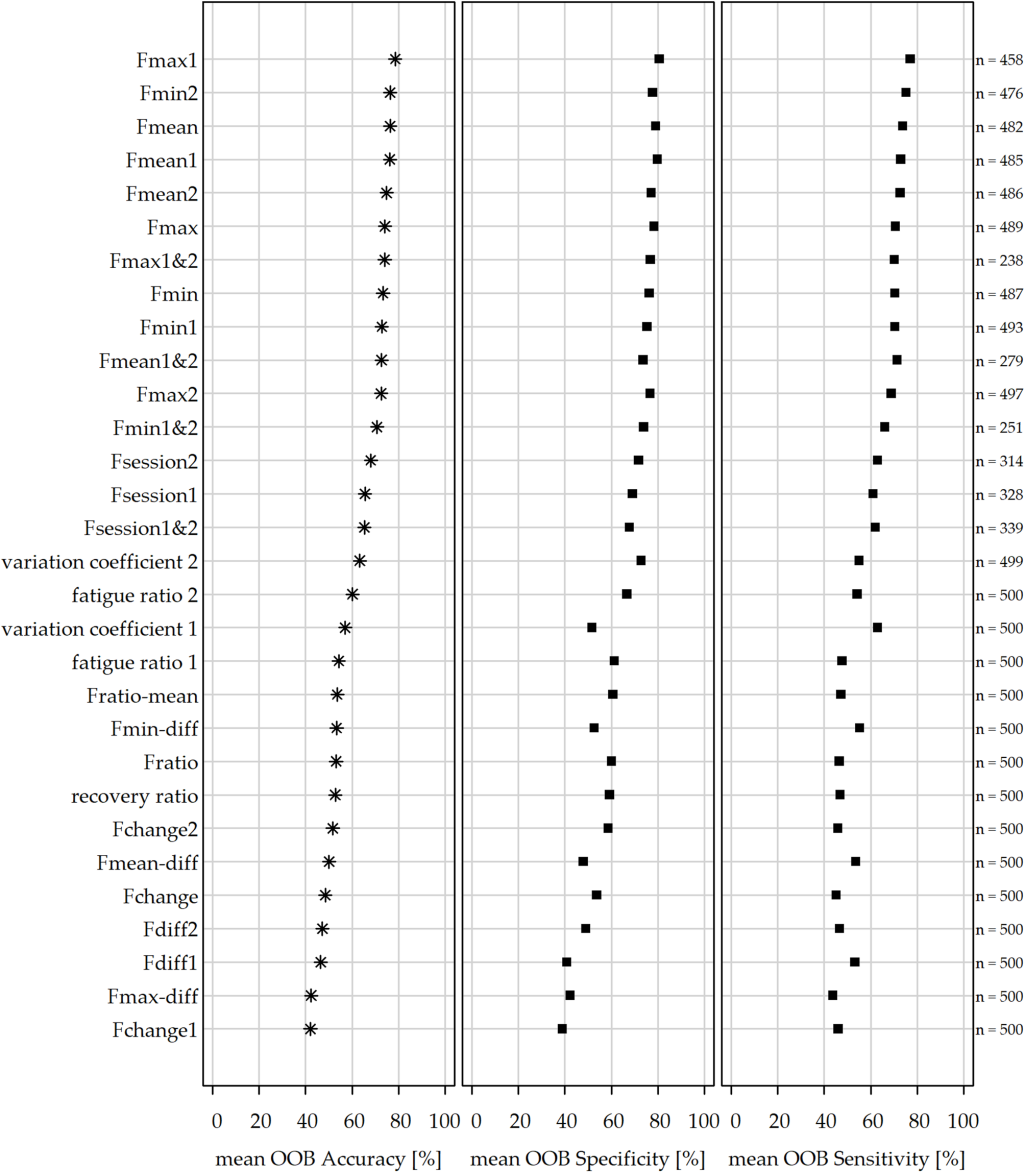
Graphical representation of bootstrap-based evaluation of predictive performance for belonging to the post-COVID syndrome (PCS) group based on hand grip strength (HGS) parameters in female hospital employees (*n* = 33; 17 PCS, 16 healthy controls). For each HGS parameter (Table 3), a separate logistic regression model including age as a covariate was fitted. Performance was evaluated using 500 bootstrap resamples and is shown as mean out-of-bag (OOB) accuracy, specificity, and sensitivity (percentages, ordered by mean OOB accuracy). Lower n reflects non-convergent models in a subset of resamples, particularly for parameters based on combined measurements. All results are listed in Supplementary Table S4.

For the estimation of the probability of belonging to the PCS group, separate logistic regression models (mixed-effects where applicable) were fitted for each HGS parameter (Table 3), including age as a covariate and restricted to female participants (*n* = 33; 17 PCS and 16 HCs). Model performance was assessed using bootstrap validation, and the resulting performance metrics are summarized in Fig. 3, with full results provided in Supplementary Table S5.

**Table 3:** Hand grip strength (HGS) parameters used for statistical analysis.

Parameter	Calculation/description	
Fmax; Fmax1; Fmax2*	Maximum HGS (kg) of all measurements, S1 and S2	
Fmin; Fmin1; Fmin2	Minimum HGS (kg) of all measurements, S1 and S2	
Fmean; Fmean1; Fmean2*	Mean HGS (kg) of all measurements, S1 and S2	
Fmean-diff	$\:Fmean1-Fmean2$	Difference between mean force in s1 and mean force in S2
Fmax-diff	$\:Fmax1-Fmax2$	Difference between maximum force in S1 and maximum force in S2
Fmin-diff	$\:Fmin1-Fmin2$	Difference between minimum force in S1 and minimum force in S2
Fdiff1	$\:Fmax1-Fmin2$	Difference between maximum and minimum force in S1
Fdiff2	$\:Fmax2-Fmin2$	Difference between maximum and minimum force in S2
Fchange1	$\:\frac{meas\:no.\:1-meas\:no.\:1.1}{\:meas\:no\:1.1}$	Relative change of HGS (kg) within the S1
Fchange2	$\:\frac{meas.\:\:no.\:2.10-meas.\:no\:2.1}{meas.\:no.\:2.1}$	Relative change of HGS (kg) within the S2
Fchange	$\:\frac{meas.\:no.\:2.10-meas.\:no.\:1.1}{meas.\:no.\:1.1}$	Relative change of HGS (kg) within both sessions
Fratio	$\:\frac{Fmean1}{Fmean2}$	Force ratio of means of both sessions
Fratio-mean	$\:\frac{\frac{meas.\:no.\:1.1}{meas.\:no.\:2.1}+\:\frac{meas.\:no.\:1.2}{meas.\:no.\:2.2}+\left(\dots\:\right)+\:\frac{meas.\:no.\:1.10}{meas.\:no.\:2.10}}{10}$ Mean of all 10 force ratios (each consists of one measurement number of S1 and the resepective measurement number of S2)	
Variation coefficient 1	$\:\frac{SD\left(Fsession1\right)}{Fmean1}$	Variation coefficient ratio of the SD of S1 and the mean of S1
Variation coefficient 2	$\:\frac{SD\left(Fsession2\right)}{Fmean2}$	Variation coefficient ratio of the SD of S2 and the mean of S2
Fatigue ratio	$\:\frac{Fmax}{Fmean}$	Fatigue ratio of the maximum value of both session and the mean of both sessions
Fatigue ratio 1*	$\:\frac{Fmax1}{Fmean1}$	Fatigue ratio of the maximum value of S1 and the mean of the S1
Fatigue ratio 2*	$\:\frac{Fmax2}{Fmean2}$	Fatigue ratio of the maximum value of S2 and the mean of S2
Recovery ratio*	$\:\frac{Fmean2}{Fmean1}$	Recovery ratio of the mean of S2 and the mean of the S1
Fmax1&2	Fmax1 and Fmax2, both considered in a mixed-effects logistic model	
Fmin1&2	Fmin1 and Fmin2, both considered in a mixed-effects logistic model	
Fmean1&2	Fmean1 and Fmean2, both considered in a mixed-effects logistic model	
Fsession1; Fsession2; Fsession1&2	All measurements of S1, S2 and both sessions combined, each considered in a mixed-effects logistic model	

*Parameters previously published and applied by Jäkel et al.^19^.

S1, session 1; S2, session 2; HGS, hand grip strength; SD, standard deviation.

Predictive performance varied across HGS parameters. The highest mean out-of-bag (OOB) accuracy was observed for Fmax1 (accuracy = 78.5%, specificity = 80.6%, sensitivity = 77.1%), followed by Fmin2 (accuracy = 76.3%, specificity = 77.8%, sensitivity = 75.2%) and mean-based parameters (Fmean, Fmean1, Fmean2; accuracies ranging from 74.8 to 76.3%). Parameters with accuracies above 70% primarily included those based on minimum, maximum, and mean force values.

Previously proposed parameters showed lower predictive performance, including fatigue ratio 2 (accuracy = 60.2%, specificity = 66.8%, sensitivity = 54.2%), fatigue ratio 1 (accuracy = 54.3%, specificity = 61.3%, sensitivity = 47.7%) and recovery ratio (accuracy = 52.8%, specificity = 59.2%, sensitivity = 46.9%).

Across models, specificity was consistently higher than sensitivity, indicating better classification of controls than PCS cases. Several parameters showed limited predictive performance, with accuracy close to chance level (~ 50%).

Models based on combined measurements (i.e., parameters combining values from both sessions within a single model) and fitted as mixed-effects logistic regression models showed lower convergence rates compared to models based on single measurements, resulting in a reduced number of evaluable bootstrap iterations for parameters such as Fmax1&2, Fmean1&2, and Fmin1&2, due to non-convergence in a subset of bootstrap samples.

### Regression models for Fmax1 and Fmin2

Both Fmax1 and Fmin2 were significantly negatively associated with the probability of belonging to the PCS group. For Fmax1, each unit increase was associated with a 20% reduction in the odds of PCS (odds ratio (OR) = 0.80, *p* = 0.014), while for Fmin2, the reduction was approximately 31% (OR = 0.69, *p* = 0.005). Age was not a significant predictor in either model (Table 4).

**Table 4:** Logistic regression models for Fmax1 and Fmin2.

		Estimate	OR	SE	z value	*p* value
*Fmax1*	*(Intercept)*	4.969		2.802	1.773	0.076
	*age*	0.007	1.007	0.038	0.187	0.852
	*Fmax1*	−0.221	0.802	0.090	−2.445	**0.014**
*Fmin2*	*(Intercept)*	4.664		2.475	1.885	0.059
	*age*	0.041	1.042	0.046	0.905	0.365
	*Fmin2*	−0.369	0.691	0.130	−2.835	**0.005**

Logistic regression models for the prediction of belonging to the post-COVID syndrome (PCS) group in female hospital employees, based on the hand grip strength (HGS) parameters Fmax1 and Fmin2, including age as a covariate (*n* = 33; 17 PCS, 16 healthy controls). Regression coefficients (estimate), corresponding odds ratios (OR), standard errors (SE), z values, and p values are reported. Negative estimates indicate lower odds of belonging to the PCS group with increasing HGS values.

### Exploratory correlation analysis

Exploratory correlation analyses based on parameters derived from minimum, maximum, and mean force values for each session showed moderate positive correlations between HGS parameters and the Bell Score (*r* = 0.317 to 0.488) and moderate negative correlations with the number of fulfilled CCC items for ME/CFS (*r* = − 0.455 to − 0.553; Supplementary Table S6).

## Discussion

HGS has been proposed as a diagnostic tool in ME/CFS^16,19^ and has more recently been applied to patients with PCS who fulfill these criteria^7,22,25^. A standardized measurement procedure has been proposed but is not yet established. Consequently, the interpretation of HGS values remains uncertain, particularly in individuals with mild to moderate PCS. In this exploratory study, HGS was assessed in HEs with PCS and HCs across repeated measurements and two sessions to investigate potential differences in force performance and fatigability. The applied linear mixed model suggests that HGS trajectories may differ between groups, although no consistent overall group effect was observed. Taken together, these findings may indicate that HGS reflects aspects of impaired muscle function in PCS; however, they should be interpreted with caution.

As demonstrated in Fig. 1, visual differences in the force of HGS in PCS and HC groups are detectable. The linear mixed model revealed significant main effects of session, measurement number, age, and sex, whereas the overall main effect of the study group was not significant. This indicates that HGS is primarily influenced by repeated testing, demographic factors, and potential practice or fatigue effects rather than PCS alone. Post-hoc analyses, however, identified specific differences between PCS and HC participants: predicted HGS was lower in PCS at the beginning and end of each session, particularly at higher measurement numbers and in older participants. Within the HC group, expected age-and sex-related differences were observed, while these effects were attenuated or absent in PCS. This context-dependent pattern may explain the absence of a global group effect and suggests that potential impairments in PCS may become apparent under repeated testing conditions rather than as a uniform reduction in strength. Sensitivity analyses confirmed that the direction of group differences remained consistent after adjustment for age and sex, whereas statistical significance was lost in the fully adjusted model. This suggests that part of the observed differences may be influenced by demographic confounding. Overall, the results indicate that PCS does not affect HGS uniformly, but rather in a context- and situation-specific manner rather than of a generalized reduction in strength.

Lower HGS values in PCS have been reported previously in studies comparing ME/CFS^19^ and PCS^26^ patients with controls. In the present analysis, HGS appeared to be lower in PCS at specific measurement points, although no consistent overall group effect was observed. Reduced functional status (Bell Score) and higher symptom burden based on CCC items in the PCS group may be in line with these observations and could reflect underlying physical impairment and fatigue. This interpretation is supported by prior studies reporting associations between lower HGS and reduced functional status, higher scores in the DePaul Symptom Questionnaire for PEM and higher fatigue scores^19,22^.

Exploratory correlation analysis using parameters derived from minimum, maximum, and mean force values across both sessions showed moderate associations between HGS parameters and clinical measures, with positive correlations for functional status (the Bell Score) and negative correlations for symptom burden based on fulfilled CCC items. These findings may suggest a relationship between reduced grip strength and clinical severity; however, given the exploratory nature of these analyses and the reliance on self-reported CCC data, they should be considered hypothesis-generating. This limitation is particularly relevant, as variability in self-reported symptom burden may influence the observed association. Nevertheless, previous studies have also reported associations between maximum and minimum HGS and clinical measures of disease severity and pain analogue scales^20^, and have suggested that maximum HGS values may relate to physical capacity^27^.

Reduced HGS has also been reported in a range of other conditions, including advanced cancer^28^, cancer-related fatigue^19^, and multiple sclerosis^20^. In the present analysis, HGS appeared to be lower in PCS at specific measurement points; however, no consistent overall group effect was observed. With regard to repeated measurements, HGS was significantly lower in the second session compared to the first in the PCS group, whereas no significant session-related differences were observed in controls. This pattern may indicate altered or delayed recovery in PCS, as previously described in ME/CFS^19^, although this interpretation should be made with caution. At the same time, both groups showed a general decline in force across measurements within each session, and no clear difference in the slopes of decline was observed. This suggests that both groups showed a similar decline in force during repeated measurements, while differences between groups may rather be related to overall performance levels or recovery between sessions. In line with this, similar HGS impairments have been reported in PCS patients with and without ME/CFS, although functional status was lower in those fulfilling ME/CFS criteria^22^.

Previous studies have assessed HGS using a limited number of repetitions, often based on three measurements^20,27^, whereas more recent studies in PCS and ME/CFS have adopted extended protocols with repeated measurements across sessions^7,22,25^. In line with these studies, we applied the protocol proposed by Jäkel et al.^19^, consisting of two familiarization attempts followed by ten consecutive measurements in two sessions. While a standardized protocol has not yet been established and evidence comparing different measurement approaches remains limited, the use of repeated measurements may provide a more comprehensive assessment of force development and fatigability, particularly in conditions characterized by variability and fluctuating symptoms. Compared to single or few measurements, this approach allows for the analysis of performance trajectories within and between sessions. Adopting a protocol consistent with recent studies was intended to facilitate comparability and to contribute to a more consistent methodological framework for future investigations of HGS in PCS and related conditions. The hand dynamometer used in this study has been previously been evaluated and shown to provide reliable measurements of HGS, and appears to correspond to the same device platform (EH101) used by Jäkel et al.^19^.

The HGS measurements showed substantial intra- and inter-individual variability, including occasional fluctuations despite a standardized protocol. To account for this variability in a relatively small dataset, a linear mixed model was applied, allowing the simultaneous consideration of fixed effects (age, sex, study group, session, and measurement number) and subject-specific random effects. The inclusion of random intercepts and slopes enabled the modeling of individual performance trajectories and contributed substantially to the explained variance, indicating that a considerable proportion of variability was attributed to between-subject differences. These differences may reflect factors not explicitly captured in the model, such as physical activity level, body composition, or individual physiological characteristics. While the inclusion of additional covariates (e.g. body mass index, as suggested by Nacul et al.^20^) might further reduce unexplained variance, the applied modeling approach allows a robust representation of overall HGS trajectories while accounting for within-subject correlations and measurement variability. In contrast to analyses based solely on absolute values, the use of model-based predictions enables the assessment of dynamic changes in HGS across repeated measurements and sessions.

In the further exploratory analysis, several HGS parameters showed a moderate ability to discriminate between PCS and control participants. Parameters derived from maximum, minimum and mean force values yielded the highest predictive performance, with Fmax1 and Fmin2 showing the most consistent results across bootstrap validation. The highest accuracy was observed for parameters reflecting maximum, minimum, and mean force values rather than the ratio-based measures describing the decline of force over time, as proposed by Jäkel et al.^19^ and subsequent studies^7,22,29^. While the linear mixed model indicated a decline in force over time, this pattern was observed in both groups, suggesting that ratio-based parameters may have limited discriminatory value due to insufficient between-group differences. These findings may suggest that reduced force capacity, particularly at the beginning (Fmax1) and later stages (Fmin2) of a measurement sequence, is potentially associated with PCS-related fatigue. The logistic regression models for Fmax1 and Fmin2 indicated a significant negative association with the probability of belonging to the PCS group, implying that higher force values were associated with a lower likelihood of PCS. However, these findings should be interpreted with caution. A relatively large number of candidate parameters (*n* = 30) were evaluated, increasing the risk of overfitting and selection bias, particularly given the limited sample size. Although bootstrap validation was applied, predictive performance remained moderate. Nevertheless, several parameters may represent promising candidates for further investigation and warrant validation in larger, independent cohorts to assess their potential diagnostic utility.

Notably, specificity was consistently higher than sensitivity across models, suggesting that HGS may be more suitable as a rule-in indicator rather than a screening tool. If confirmed beyond this exploratory analysis, HGS could therefore serve as a supportive adjunct to clinical assessment, with clinical presentation remaining the primary basis for diagnosis rather than HGS functioning as an independent diagnostic test.

It should further be noted that parameters based on minimum or maximum force values may be particularly sensitive to variability, as they reflect extreme values within a sequence of measurements and are therefore more susceptible to the influence of outliers. In contrast, parameters derived from multiple observations (e.g. mean values or variability-based metrics) may provide more stable estimates, although they are not entirely robust to extreme values. More generally, models relying on absolute force values may have limited generalizability to other populations, as they reduce dynamic performance patterns to single summary measures and may be influenced by differences in age distribution, physical condition, or fatigue levels, even under standardized testing conditions. Overall, the present findings suggest that certain HGS-derived parameters may carry potential for characterizing PCS-related functional impairment. However, their clinical utility for individual-level prediction remains uncertain and requires confirmation in larger, independent cohorts.

Vaccination status and viral variants may have influenced the observed findings and should be considered when interpreting the results. The control group showed a higher vaccination rate, and vaccination has been associated with a reduced risk of developing PCS and potentially milder symptom courses^30^. In addition, the PCS cohort was infected during the early phase of the pandemic, when wild-type SARS-CoV-2 and the Alpha variant predominated, which have been linked to a higher risk of long COVID compared to later variants^3^. Furthermore, 42% of the PCS group reported at least one reinfection, which has been associated with a lower likelihood of recovery^3^. However, it remains unclear to what extent these factors directly influence muscle strength or fatigability. Additionally, the long interval between infection and assessment may have contributed to heterogeneous disease trajectories and increased the likelihood of additional confounding factors that could not be fully controlled for.

The control group was classified based on routine occupational testing and symptom monitoring during the pandemic; however, prior asymptomatic SARS-CoV-2 infections cannot be fully excluded in the absence of serological testing. Participants were further characterized using the CCC to assess symptoms consistent with ME/CFS, although a clinical diagnosis cannot be fully excluded despite being unlikely in this group. The PCS group comprised participants with and without ME/CFS, reflecting the heterogeneity of the condition. Comorbidities were present mostly only in the PCS group but were reported to be clinically stable under ongoing treatment at the time of assessment. While their overall frequency was limited, a potential influence on HGS cannot be entirely excluded. Potential pathomechanisms within this cohort, including autoantibodies against G-protein coupled receptors, Epstein-Barr virus reactivations, and chronic inflammatory processes, have been described previously^10^. The heterogeneity may have attenuated subgroup-specific effects; however, it is consistent with the current understanding of PCS as a multisystemic condition with incompletely understood mechanisms^3,31^. Participants were recruited from a previously characterized cohort of HEs reporting persistent fatigue, without further selection or exclusion within this subgroup. This approach was intended to reflect a broad spectrum of PCS presentations. The predominance of female participants is in line with previous observations suggesting a higher risk of PCS in women^32^. In addition, all PCS participants were unvaccinated at the time of initial infection and were likely infected with early SARS-CoV-2 variants, which may have influenced disease manifestation^1^. The cohort represents a moderately affected PCS population (mean Bell Score 65), in contrast to more severely affected cohorts (median Bell Scores of 40 and 50) reported elsewhere, where lower functional status has been associated with more pronounced HGS impairment^22,25^. These differences should be interpreted in light of the respective study populations, as previous studies were conducted in specialized centers with potentially more severely affected patients, whereas the present study is based on a predefined occupational cohort.

Pathophysiological explanations for lower HGS in PCS might include hypoperfusion of skeletal muscles, as demonstrated in a study using MRIs showing higher sodium content in muscles of ME/CFS patients compared to HCs. The correlation of HGS with higher sodium content suggests impaired function of ion transport in ME/CFS caused by hypoperfusion^33^. Current knowledge on the pathophysiology of skeletal muscle in ME/CFS and post-COVID ME/CFS, respectively, suggests skeletal muscle damage and mitochondrial dysfunction caused by increased intracellular sodium concentration, leading to calcium overload. If sustained mitochondrial dysfunction induces an ongoing vicious cycle, this might explain PEM. Infection-triggered mechanisms such as endothelial dysfunction, inflammation, and autoantibodies also cause circulatory disturbances and hypoperfusion of muscles^34^. In the context of PEM in PCS diminished exercise capacity of skeletal muscles was observed, alongside severe exercise-induced myopathy^35^.

The utility of HGS as a diagnostic marker for a specific disease appears to be limited, particularly when HGS values are compared to heterogeneous samples rather than well-defined HC groups. However, HGS has been shown to be associated with clinical scores^19,20,22^, laboratory values^19,36^, as well as maximum work rate^27^. Nevertheless, these correlations offer a valuable opportunity to objectively quantify and track clinical changes over time, whether in response to a specific intervention or during routine longitudinal monitoring, anchored by an initial baseline measurement of HGS. This approach was illustrated in a small-scale study involving patients with ME/CFS, in which creatine supplementation led to a significant increase in HGS^37^.

### Limitations

This study has several limitations that should be considered when interpreting the findings. First, the absence of serological testing prevents definitive exclusion of prior SARS-CoV-2 infection in the control group. Although classification was based on repeated occupational PCR and antigen testing as well as symptom history, asymptomatic infections cannot be ruled out. Such potential misclassification would likely bias the results towards the null and attenuate observed group differences. Second, the CCC were applied as a self-reported screening tool in the control group without clinical evaluation. While a diagnosis of ME/CFS in this group is unlikely given the required symptom duration, misclassification of symptom burden cannot be excluded and should be considered when interpreting group comparisons. Third, although reported comorbidities were clinically stable and under ongoing treatment at the time of assessment, their potential influence on HGS was not systematically controlled for and may represent a source of residual confounding. In addition, relevant variables such as body mass index or perceived fatigue scores were not systematically available and could therefore not be included in the analysis. Fourth, the relatively small sample size and the lack of matching between groups, particularly with respect to age and sex, together with the imbalance in sex distribution, limit statistical power and may introduce residual confounding. In addition, the sample size did not allow for detailed subgroup analyses, particularly with respect to participants with and without ME/CFS. As a result, subgroup-specific effects could not be reliably assessed. The exploratory nature of the analyses, including the evaluation of multiple HGS parameters, further increases the risk of overfitting and limits the robustness of predictive findings. Finally, the cohort was derived from a predefined group of HEs with persistent fatigue, which may introduce selection bias and limit comparability to other PCS populations. Overall, the findings should be considered hypothesis-generating and require confirmation in larger, independent, and well-characterized cohorts.

## Conclusion

HGS represents a simple, inexpensive and reproducible measure that may provide insights into functional impairment in PCS. In this exploratory study, HGS tended to be lower in PCS compared to HCs at specific measurement points, while no consistent overall group effect was observed. In addition, HGS was reduced in the second session in PCS but not in controls, which may indicate altered or delayed recovery following repeated exertion. Exploratory analyses identified certain HGS-derived parameters, particularly those based on maximum, minimum, and mean force values, as potentially informative for characterizing PCS-related differences; however, their predictive performance was moderate and should be interpreted with caution. Overall, HGS may serve as a supportive adjunct in the clinical assessment of PCS, particularly as a rule-in indicator, but its utility as a screening or standalone diagnostic tool appears limited. Further validation in larger, standardized cohorts is required before clinical use.

## Methods

### Study design

Observational case-control study analyzing HGS of HEs with PCS following a SARS-CoV-2 infection and HC of three medical centers, belonging to one hospital in Cologne (Germany). The study was conducted as an exploratory pilot investigation. This research is part of the HALE (*Health Care Workers Affected by Long COVID and Exhaustion/Fatigue*) study 2022.

### Settings and subjects

**Fig. 4:**
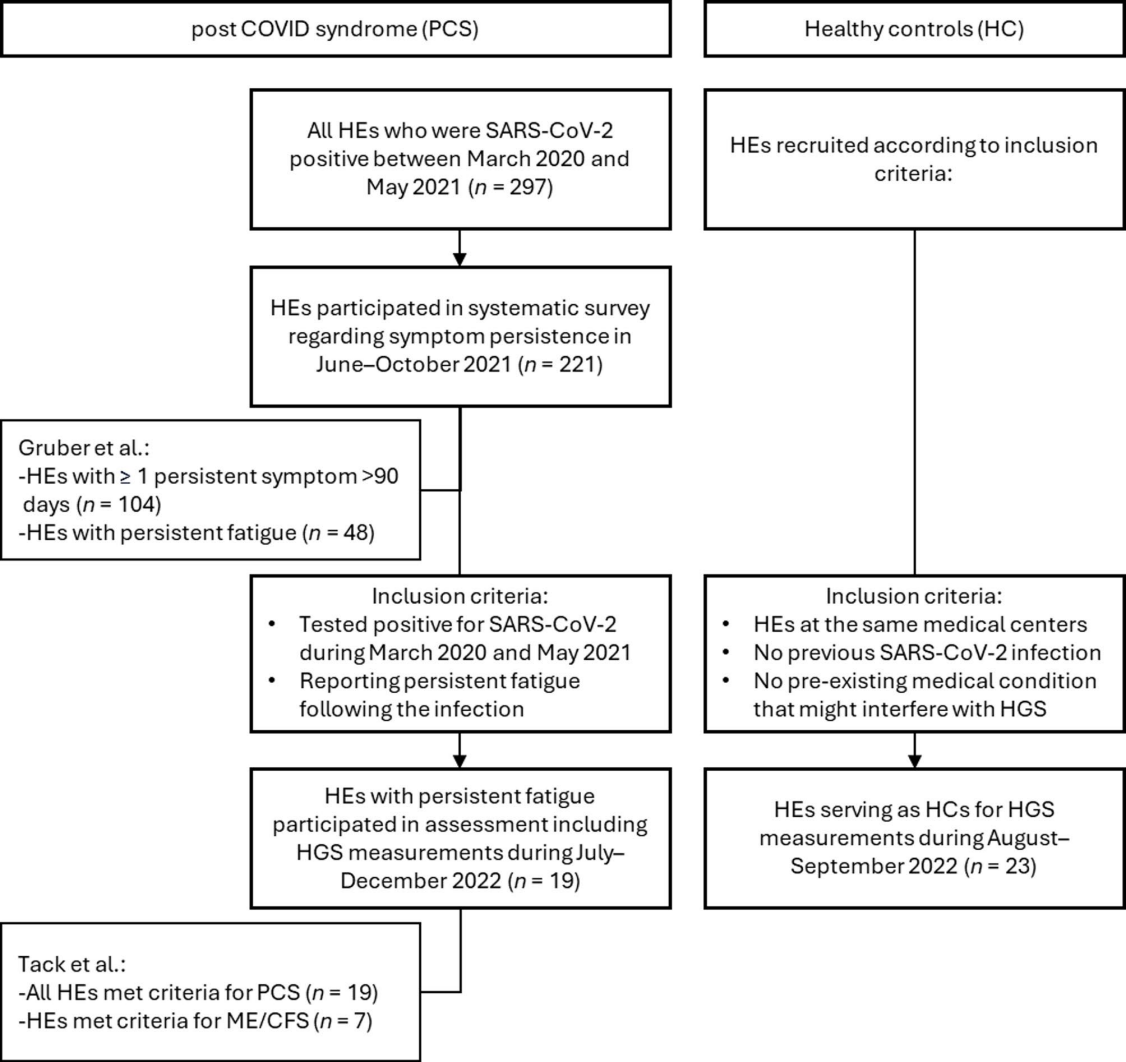
Flowchart illustrating the selection of participants. The post-COVID syndrome (PCS) group consisted of SARS-CoV-2-positive hospital employees (HE) who initially participated in a systematic survey conducted by Gruber et al.^38^. Participants who reported persistent fatigue were subsequently invited to a follow-up assessment including hand grip strength (HGS) measurements^10^. The PCS group was compared with healthy controls (HC), who were HEs from the same medical centers.

The PCS group was drawn from a cohort of 221 HEs with a confirmed SARS-CoV-2 infection between March 2020 and May 2021. A systematic follow-up survey of this cohort, conducted by Gruber et al. between June and October 2021, showed that a substantial proportion of HEs (*n* = 48) still reported persistent fatigue^38^. Of these, 19 participated in a comprehensive follow-up assessment, including HGS measurements, between July and December 2022. Inclusion criteria were persistent fatigue and a documented SARS-CoV-2 infection within the specified time frame while working as a HE at the participating medical centers^10^. A flowchart illustrating participant selection is provided in Fig. 4.

The PCS cohort has been described previously in detail. Briefly, persistent fatigue was the main symptom following SARS-CoV-2 infection, while at least 84% of participants additionally reported insomnia, concentration disorders, palpitations, memory disorders, breathlessness, limb weakness and anxiety^10^. PCS was diagnosed according to the WHO criteria^4^, and ME/CFS was diagnosed according to the CCC^39^, applied in the German translation provided by the Charité Fatigue Center^40^. A standardized medical assessment was performed to evaluate participants’ medical status and exclude alternative explanations for the symptoms. This assessment included physical examination, laboratory testing and referral to relevant hospital departments when indicated^10^.

The present study represents an additional analysis focusing on HGS in comparison with HC. The control group comprised 23 HEs from the same medical centers who reported no known prior SARS-CoV-2 infection based on testing history and symptom assessment and considered themselves physically fit. Participants were also screened for pre-existing medical conditions. HGS measurements in the control group were conducted between August and September 2022.

All participants were at least 18 years of age and provided written informed consent. Participants were excluded only if they withdrew their consent; no additional exclusion criteria were applied. No participants withdrew or were excluded during the HGS assessment or subsequent data analysis.

### Assessment and data collection

The study group (PCS) was assessed during an outpatient appointment as previously described^10^. Participants were screened for symptoms consistent with ME/CFS using the CCC, where applicable, and were further evaluated to exclude differential diagnoses as previously reported^10^.

The control group completed a standardized questionnaire including general characteristics such as age, sex, occupation, and COVID-19 vaccination status (number and type of doses). In addition, controls participants completed the CCC questionnaire as a self-reported screening tool to assess the presence and number of symptoms associated with ME/CFS. Given that the CCC require a minimum symptom duration of six months, a formal diagnosis of ME/CFS in this group was considered unlikely. The questionnaire was therefore used to characterize baseline symptom reporting and to provide a reference for comparison with the PCS group, rather than to establish or exclude a clinical diagnosis. Participants were able to seek clarification if needed during completion.

For both groups, functional status was assessed using the Bell Score, a scale ranging from 0 (mostly bedridden) to 100 (healthy) in increments of 10^41^.

From the beginning of the pandemic until the time of assessment, all HEs were screened for SARS-CoV-2 infections in case of contact to infected individuals, acute symptoms, or after spending time in high-risk areas using hypopharyngeal swabs analyzed by polymerase chain reaction (PCR)^42^. Additionally, from April 2021 until the assessments, all HEs had the opportunity to take up to two SARS-CoV-2 antigen tests per week; these were required for unvaccinated individuals. These measures support that the control group had no documented SARS-CoV-2 infection during the observation period; however, asymptomatic infections cannot be excluded. The PCS group was additionally tested at the time of HGS measurement to exclude an acute SARS-CoV-2 infection.

The hand dynamometer used in this study (GRIPX EH101) appears to correspond to the same device platform (EH101) that is commonly marketed under different brand names, including CAMRY EH101. This device has been evaluated in previous studies and demonstrated good reliability and validity^43^; however, validation data for this specific model in PCS populations are limited. The device shows the highest force measured during a pull in kg. HGS measurement comprises two sessions with ten consecutive measurements each and a 60 min interval between sessions. For the measurements, participants were asked to sit upright with the forearm positioned on the table in front, elbow flexed at 90 degrees. Participants were asked to use their dominant hand and pull the handle with maximum possible force. Each participant did two practice attemps to get familiar with the device. Participants tried to pull with maximum force for 3 s and stopped for 5 s before the next try. During the procedure a trained observer supervised the execution, gave directions for each repetition and motivated the particpants to reach their maximum force^19^.

As described by Jäkel et al., maximum force (Fmax) and mean force (Fmean) were determined for each session. Fatigability was assessed for both sessions using the fatigue ratio (Fmax/Fmean), with higher values indicating a stronger decline in force during a session. Recovery was assessed using the recovery ratio (Fmean2/Fmean1), with lower values indicating impaired recovery^19^.

For the analysis of potential parameters to compare study and control groups, a set of additional HGS-derived parameters was explored, as listed in Table 3. These parameters were considered exploratory and were evaluated to identify potential markers of group differences.

### Data analysis

Descriptive statistics for the study population were performed. Female and male results were analyzed separately due to sex-related differences in HGS.

Study group (PCS vs. HC) was included as the main exposure variable. A linear mixed model was fitted to predict HGS based on session, study group, and measurement number, including relevant interactions. Age and sex were included as covariates in interaction with study group, and random intercepts and slopes were specified for each participant.

Global F-tests were performed to assess the significance of fixed effects and interactions. For factors showing significant differences, pairwise post-hoc t-tests with 95% confidence intervals (95% CI) were conducted. P-values from post-hoc tests were Bonferroni-adjusted to control for multiple comparisons^44^.

To assess the potential confounding effects of age and sex on the association between study group and HGS, sensitivity analyses were performed using different specifications of the mixed linear model. Specifically, four model specifications were fitted: (1) an unadjusted model, (2) a model adjusted for sex, (3) a model adjusted for age, and (4) a fully adjusted model including both age and sex.

All models included the same fixed and interaction terms as specified in the primary analysis, with age and sex added as covariantes where appropriate. This approach allowed for the evaluation of the stability of the estimated effects of study group across varying levels of adjustment.

Marginal predictions of the probability of belonging to the PCS group versus HC were modeled using logistic regression models^44^^45^,, including age as a covariate. Sex was not included due to the small number of male participants in the PCS group. For each HGS parameter, a separate regression model was constructed. For parameters with repeated measurements per participant (Fsession1&2, Fsession1, Fsession2, Fmean1&2, Fmin1&2, Fmax1&2), mixed-effects logistic regression models with random subject effect were applied^46^. For all other HGS parameters, standard logistic regression models were used. To assess the predictive performance of each model, a bootstrap validation approach was applied. A total of 500 bootstrap samples (training datasets) of size *n* = 33 were drawn. For each bootstrap sample, the corresponding out-of-bag (OOB) sample, consisting of participants not included in the bootstrap sample, was used as test data. For each model, the following steps were repeated across all bootstrap iterations: (1) the model was fitted to the bootstrap training data; (2) the optimal classification threshold was determined using the Youden index^47^; in cases of multiple optimal thresholds, the threshold yielding higher specificity was selected; and (3) predictions were generated for the corresponding OOB sample, and performance metrics including accuracy, sensitivity, and specificity were calculated.

An exploratory correlation analysis was performed to assess association between HGS parameters and clinical scores (Bell Score and the number of fulfilled items of the CCC for the diagnosis of ME/CFS) using Pearson’s correlation coefficients.

P-values < 0.05 were considered statistically significant. All analyses were performed using the statistical software R (version 4.3.1; R Core Team, 2023 (Austria))^48^ with the additional packages “emmeans”^49^, “ImerTest”^46^, “MuMIn”^50^, and “pROC”^47^. The statistical code used for the analyses is provided as Supplementary Code.

## Supplementary Information

Below is the link to the electronic supplementary material.

Supplementary Material 1

Supplementary Material 3

## Data Availability

The datasets generated during and/or analyzed during the current study are available from the corresponding author on reasonable request.
