# Supplementary material for: Interpreting hand grip strength in hospital employees with post-COVID syndrome compared to non-infected controls: a case-control study
Source: Sci Rep. 2026 May 9;16:14725. doi: 10.1038/s41598-026-51666-w (PMC13161236; doi:10.1038/s41598-026-51666-w)
Supplement: Supplementary file 1 — Supplementary Material 1 [file 41598_2026_51666_MOESM1_ESM.pdf]

SUPPLEMENTARY MATERIAL

Supplementary Table S1 Fixed effects of the linear mixed model

	estimate	*SE*
(Intercept)	25.103	5.719
session = 2	−0.946	0.507
PCS (vs. HC)	−3.863	7.854
measurement number	−0.460	0.095
age	0.037	0.121
sex = male	32.312	7.762
session = 2 x PCS (vs. HC)	−0.691	0.754
session = 2 x measurement number	0.146	0.082
PCS (vs. HC) x measurement number	0.033	0.141
PCS (vs. HC) x age	−0.069	0.161
PCS (vs. HC) x sex = male	−7.160	4.908
age x sex = male	−0.421	0.174
session = 2 x PCS (vs. HC) x measurement number	−0.124	0.122

Estimated fixed effects of the linear mixed model analyzing hand grip strength (HGS), including main effects and interaction terms for session, study group (post-COVID syndrome (PCS) vs. healthy controls (HC)), measurement number, age, and sex. Regression coefficients (estimate) and corresponding standard errors (SE) are reported. The model includes random intercepts and random slopes at the subject level.

Supplementary Table S2 Global F-test for linear mixed model

model term	*df1*	*df2*	*F.ratio*	*p.value*
Session	1	752.000	15.158	**<0.001**
Study group (PCS vs. HC)	1	39.460	3.552	0.067
Measurement number	1	40.000	39.698	**<0.001**
Age	1	35.000	5.476	**0.025**
Sex	1	35.000	15.741	**<0.001**
Session x study group	1	752.000	2.149	0.143
Session x Measurement number	1	752.000	1.913	0.167
Study group x Measurement number	1	40.000	0.052	0.820
Study group x Age	1	35.000	0.175	0.678
Study group x Sex	1	35.000	2.014	0.165
Age x Sex	1	35.000	5.542	**0.024**
Session x Study group x Measurement number	1	752.000	1.045	0.307

Global F-tests from the linear mixed model predicting hand grip strength, showing manin and interaction effects of session, measurement number, study group, age, and sex. Degrees of freedom (df1, df2), F-ratios and p-values are reported for all effects, with significant effects highlited in bold.

Supplementary Table S3 Comparison of slopes within a linear mixed model for the prediction of hand grip strength

**Comparison of the slope of force prediction for both study groups in relation to the sessions**								
session	***study group***	*estimate*	*SE*	*df*	*2.5%*	*97.5%*	*t.ratio*	*p.value*
1	**PCS - HC**	0.033	0.141	60.166	-0.291	0.357	0.234	1
2	**PCS - HC**	-0.091	0.141	60.166	-0.416	0.233	-0.647	1
**Comparison of the slope of force prediction for both sessions in relation to both study groups**								
study group	***session***	*estimate*	*SE*	*df*	*2.5%*	*97.5%*	*t.ratio*	*p.value*
HC	**2 - 1**	0.146	0.082	752.000	-0.037	0.330	1.788	0.148
PCS	**2 - 1**	0.022	0.090	752.000	-0.180	0.224	0.244	1.000
**Comparison of the slope of force prediction for both study groups**								
study group		*estimate*	*SE*	*df*	*2.5%*	*97.5%*	*t.ratio*	*p.value*
**PCS - HC**		-0.069	0.166	35.000	-0.406	0.267	-0.419	0.678
**Comparison of the slope of force prediction for sex**								
sex		*estimate*	*SE*	*df*	*2.5%*	*97.5%*	*t.ratio*	*p.value*
**male - female**		-0.421	0.179	35.000	-0.783	-0.058	-2.354	**0.024**

Post-hoc-tests comparing predicted hand grip strength (HGS) between hospital employees (HE) with post-COVID syndrome (PCS, n=19) and HEs serving as healthy controls (HC, n=23) using a linear mixed model. Compared slopes of regression lines for specific variables are highlighted in bold and correspond to the first column of the table, indicating the factors from the model. The 95% confidence intervals for the estimates are provided at the 2.5% and 97.5% limits. All p-values were Bonferroni-adjusted. SE, standard error; df, degrees of freedom.

Supplementary Table S4 Sensitivity analysis using different specifications of the linear mixed model

	Unadjusted Model	*No age*	*No sex*	*Full Model*
Study group (PCS vs. HC)	-11.27 (2.92)**	-7.09 (2.48)**	-18.89 (10.28)	-3.86 (7.85)
Study group x Session	-0.69 (0.75)	-0.69 (0.75)	-0.69 (0.75)	-0.69 (0.75)
Study group x Measurement number	0.03 (0.14)	0.03 (0.14)	0.03 (0.14)	0.03 (0.14)
Study group x Session : Measurement number	-0.12 (0.12)	-0.12 (0.12)	-0.12 (0.12)	-0.12 (0.12)
Session	-0.95 (0.51)	-0.95 (0.51)	-0.95 (0.51)	-0.95 (0.51)
Measurement number	-0.46 (0.09)**	-0.46 (0.09)**	-0.46 (0.09)**	-0.46 (0.09)**
Session x Measurement number	0.15 (0.08)	0.15 (0.08)	0.15 (0.08)	0.15 (0.08)
Sex	-	15.23 (2.6)**	-	32.31 (7.76)**
Sex x Study group	-	-10.89 (5.02)	-	-7.16 (4.91)
Age	-	-	-0.25 (0.13)	0.04 (0.12)
Age x Study group	-	-	0.18 (0.21)	-0.07 (0.16)
Sex x Age	-	-	-	-0.42 (0.17)

Results of sensitivity analyses using different specifications of the linear mixed model to assess the association between study group (post-COVID syndrome (PCS) vs. health controls (HC)) and hand grip strength. Four model specifications are presented: unadjusted, adjusted for sex only (“No age”), adjusted for age only (“No sex”), and fully adjusted for both age and sex (“Full model”). Values represent regression coefficients with standard errors in parentheses. Session and measurement number were included as repeated-measures factors. Interaction terms between study group, session and measurement number are shown where applicable. Sex and age were included as covariates in the respective models. *p < 0.0.5; **p < 0.01; ***p < 0.001.

Supplementary Table S5 Bootstrap-based predictive performance of HGS parameters for PCS group membership

HGS Statistic	*n*	*mean OOB Accuracy [%]*	*mean OOB Specificity [%]*	*mean OOB Sensitivity [%]*
Fmax1	458	78.527	80.594	77.075
Fmin2	476	76.327	77.811	75.197
Fmean	482	76.301	79.124	73.798

Fmean1	485	76.216	79.856	72.904
Fmean2	486	74.831	77.185	72.727
Fmax	489	74.103	78.294	70.693
Fmax1&2	238	73.996	76.842	70.177
Fmin	487	73.298	76.259	70.385
Fmin1	493	72.773	75.353	70.400
Fmean1&2	279	72.680	73.696	71.390
Fmax2	497	72.496	76.629	68.926
Fmin1&2	251	70.695	73.898	66.085
Fsession2	314	68.043	71.788	62.976
Fsession1	328	65.506	69.043	61.025
Fsession1&2	339	65.270	67.777	62.023
variation coefficient 2	499	63.286	72.863	55.088
fatigue ratio 2	500	60.151	66.753	54.183
variation coefficient 1	500	57.006	51.637	62.945
fatigue ratio 1	500	54.262	61.277	47.709
Fratio-mean	500	53.621	60.695	47.277
Fmin-diff	500	53.317	52.665	55.277
Fratio	500	53.126	60.138	46.563
recovery ratio	500	52.760	59.230	46.853
Fchange2	500	51.654	58.591	45.858
Fmean-diff	500	50.095	47.903	53.617
Fchange	500	48.422	53.735	45.194
Fdiff2	500	47.198	49.083	46.583
Fdiff1	500	46.433	40.884	53.259
Fmax-diff	500	42.364	42.352	43.804
Fchange1	500	42.016	38.878	46.030

Bootstrap-based evaluation of predictive performance for belonging to the post-COVID syndrome (PCS) group based on hand grip strength (HGS) parameters in female hospital employees (n = 33; 17 PCS, 16 healthy controls). For each HGS parameter (Table 4), a separate logistic regression model (mixed-effects where applicable) including age as a covariate was fitted. Performance was evaluated using 500 bootstrap resamples and reported as mean out-of-bag (OOB) accuracy, specificity, and sensitivity (expressed as percentages and ordered by mean OOB accuracy). Lower n reflects non-convergent models in a subset of resamples, particularly for models incorporating combined measurements (i.e.,

parameters combining values from both sessions within a single model) that were fitted as mixed-effects logistic regression models.

Supplementary Table S6 Exploratory correlation analysis of selected hand grip strength (HGS) parameters and clinical scores

parameter	*scores*	*Pearson correlation*
Fmin1	Bell Score	0.402
	Total points CCC	−0.503
Fmin2	Bell Score	0.488
	Total points CCC	−0.553
Fmean1	Bell Score	0.366
	Total points CCC	−0.491
Fmean2	Bell Score	0.462
	Total points CCC	−0.519
Fmax1	Bell Score	0.317
	Total points CCC	−0.455
Fmax2	Bell Score	0.390
	Total points CCC	−0.469

Exploratory Pearson correlation analysis between selected HGS parameters and clinical scores, including Bell Score and the number of fulfilled items of the Canadian Consensus Criteria (CCC) for ME/CFS. Parameters shown represent a subset with comparatively high predictive accuracy and were calculated separately for the first and second session. Pearson correlation coefficients (r) are reported for all associations.

Supplementary Figure S1 Model performance

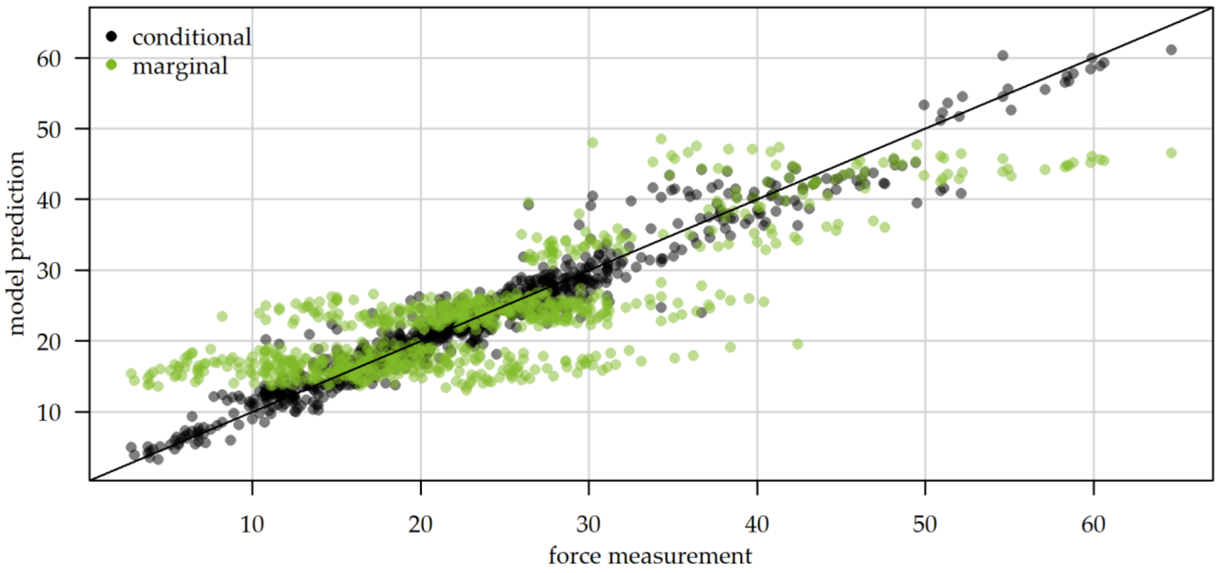

Supplementary Figure S1 Model prediction and observed force values of a linear mixed model: marginal predications (without random effects, RMSE=6.084) and conditional predications (with random effects, RMSE=2.401). Proportion of fixed effects are 62.1%, random effects are 32.2%, explaining 94.3% of total variance (conditional R^2=0.943) with remaining residuals of 5.7%.
